# Impact of Dietary Egg Yolk IgY Powder on Behavior, Meat Quality, Physiology, and Intestinal *Escherichia coli* Colonization of Broiler Chicks

**DOI:** 10.3389/fvets.2022.783094

**Published:** 2022-03-29

**Authors:** Ibrahim F. Rehan, Ahmed F. Rehan, Ahmed F. Abouelnaga, Mohamed A. Hussein, Waleed R. El-Ghareeb, Nesreen Z. Eleiwa, Asmaa Elnagar, Gaber E. Batiha, Mohamed A. Abdelgawad, Mohammed M. Ghoneim, Amin A. Hafiz, Hossam E. Gadallah, Shereen El. Abdel-Hamid, Gamal R. Hasab El-Naby, Barbara M. Benowitz, Mohamed A. Maky

**Affiliations:** ^1^Department of Husbandry and Development of Animal Wealth, Faculty of Veterinary Medicine, Menofia University, Shibin Alkom, Egypt; ^2^Department of Food Control, Faculty of Veterinary Medicine, Zagazig University, Zagazig, Egypt; ^3^Department of Husbandry and Development of Animal Wealth, Faculty of Veterinary Medicine, Mansoura University, Mansoura, Egypt; ^4^Department of Public Health, College of Veterinary Medicine, King Faisal University, Al-Ahsa, Saudi Arabia; ^5^Department of Food Hygiene, Animal Health Research Institute, Agriculture Research Center, Giza, Egypt; ^6^Department of Biochemistry, Faculty of Veterinary Medicine, Zagazig University, Zagazig, Egypt; ^7^Department of Pharmacology and Therapeutics, Faculty of Veterinary Medicine, Damanhour University, Damanhour, Egypt; ^8^Department of Pharmaceutical Chemistry, College of Pharmacy, Jouf University, Sakaka, Saudi Arabia; ^9^Department of Pharmacy Practice, Faculty of Pharmacy, AlMaarefa University, Ad Diriyah, Saudi Arabia; ^10^Department of Clinical Nutrition, Faculty of Applied Medical Sciences, Umm Al-Qura University, Mecca, Saudi Arabia; ^11^Department of Clinical Pathology, Faculty of Veterinary Medicine, Mansoura University, Mansoura, Egypt; ^12^Department of Veterinary Public Health, Faculty of Veterinary Medicine, Zagazig University, Zagazig, Egypt; ^13^Tanta Provincial Laboratory, Animal Health Research Institute, Tanta, Egypt; ^14^Department of Psychology, Gettysburg College, Gettysburg, PA, United States; ^15^Department of Food Hygiene and Control (Meat Hygiene), Faculty of Veterinary Medicine, South Valley University, Qena, Egypt

**Keywords:** immunoglobulin Y, broiler, performance, behavior, *E. coli*, meat quality

## Abstract

The current study investigated the impact of different concentrations of purified egg yolk immunoglobulin Y (IgY) supplemental food on the growth performance, behaviors, cecal contents of *Escherichia coli*, and the meat quality of broiler chicks. Four dietary groups were given to 180 female Ross broiler chicks at random (*n* = 45 for each). The control group was fed a standard diet only, whereas the other three experimental groups were fed the same basic diet supplemented with 1,500, 3,000, and 4,000 μg/ml IgY for a duration of 42 days. Significant greater behavioral activities, including, feeding, drinking, and dust bathing (*p* < 0.05), in the birds fed 4,000 μg/ml of IgY compared to the control group were observed. Greater weight gains of the crop, proventriculus, gizzard, and intestine (*p* < 0.05) were observed for broiler chicks fed 4,000 μg/ml of IgY when compared to the control group. After 3 weeks of feeding, the groups fed 3,000 and 4,000 μg/ml IgY had significant lower *E. coli* counts in the muscle and cecal contents (*p* < 0.05) when compared to the control group. Moreover, dietary supplementation with 4,000 μg/ml IgY in the third week and 3,000 μg/ml IgY in the sixth week resulted in greater weight gain (*p* < 0.01) when compared to the control group. Also, at week 3, chicks fed 4,000 μg/ml of IgY had a lower feed conversion ratio (FCR) when compared to the control group (*p* < 0.05). At week 6, chicks fed 3,000 μg/ml of IgY had lower FCR than the control (*p* < 0.05). The circulating heterophile/lymphocyte ratio was simply altered in birds fed variable IgY concentrations (1,500, 3,000, and 4,000 μg/ml), with no significant differences compared to the control group due to the individual resistance of each bird to physiological stress. The addition of 4,000 μg/ml IgY to the diet enhanced the nutritive value of meat, including protein, fat, and ash content (*p* < 0.05). Our study concluded that dietary supplementation of 3,000 and/or 4,000 μg/ml IgY improved the growth rates, behavioral activities, intestinal health indices, and meat quality of broiler chicks.

## Introduction

During the last decade, researchers have worked to enhance the hygienic conditions of broiler farms. This would be possible by moving away from using antimicrobial drugs, which are often found in poultry products such as meat and eggs as harmful residues, negatively affecting the health of human beings (1). Furthermore, owing to the contamination of carcasses during evisceration and insufficient poultry raising circumstances, the prevalence of intestinal colonization in chickens caused by the pathogenic *Escherichia coli* is frequently common, presenting an elevated danger to humans (2, 3). Egg yolk immunoglobulin Y (IgY) is a safe and effective alternative to drugs and does not induce allergic responses. This is due to the unique structure of the IgY fragment crystallization (FC) region, which prevents the Fc receptors from attaching to immune cells (4–7). IgY has attracted a lot of interest in recent years since it is simple to make in large quantities and is both cost-effective and safe (8). Newly hatched chicks receive maternal antibodies from their dams needed for egg protection (9). On the day 7 after hatching, the IgY is reduced as soon as the bird starts developing antibodies (10). Hen egg yolk has 8–20 mg/ml of IgY (11). Recently, we have revealed that broilers fed a novel combination of IgY and probiotics improved their behavioral activities and immunity responses (7). Furthermore, using this combination improved the meat quality characteristics and nutritional value (12). In addition, we determined that 1,500 μg/ml IgY had a bacteriocidal effect on Gram-positive bacteria; however, we could not evaluate which concentration(s) of IgY might be effective in killing Gram-negative bacteria. There is a necessity to adjust the effective concentration in order to kill Gram-negative bacteria. Owing to the beneficial role of IgY, we were curious to study the effects of various concentrations of IgY powder mixed in ration on the bird's behavior, performance and on the physiological parameters, meat nutritive value, and microbiological load of the meat, notably *E. coli* contamination. Our study is ultimately aimed at producing nutritional and safe meat for human consumption.

## Materials and Methods

### Study Design, Animals, and Management

**Figure 1:**
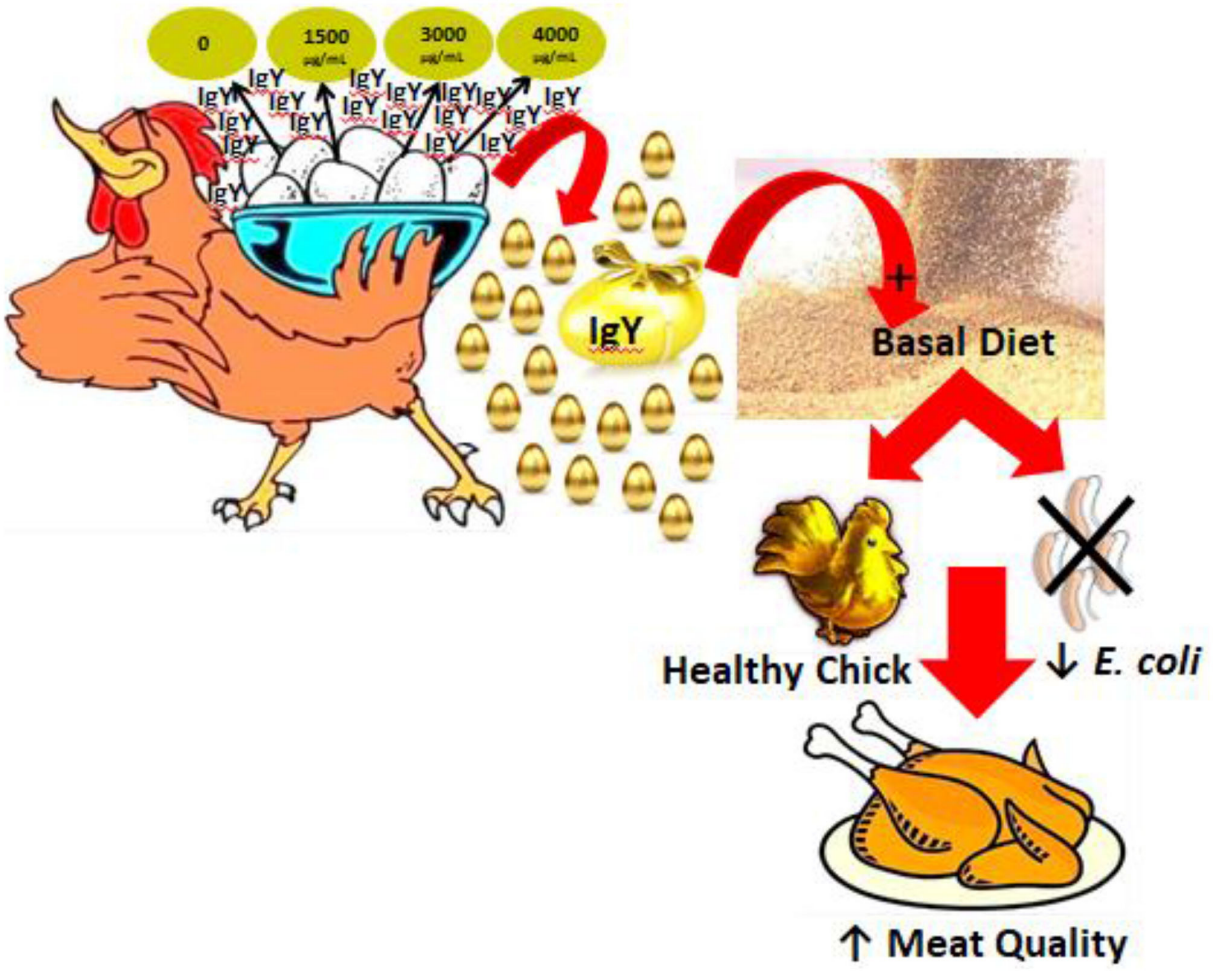
Schematic cartoon of the study strategy designed using Biorender.com.

One hundred and eighty broiler chicks (female Ross strain, 1.3 ± 0.21 days old, 48.1 ± 0.2 g) were obtained from the Egyptian Nutrivet Animal Health Company. They were randomly divided into four equivalent groups (*n* = 45) with three replicates and handled up to day 42. Multiple concentrations (0, 1,500, 3,000, and 4,000 μg/ml) of IgY were used. The schematic cartoon in Figure 1 shows the study design of the four experimental groups. The first group, serving as the control, received the basal ration, whereas the second, third, and fourth groups were fed different concentrations of IgY powder (1,500, 3,000, and 4,000 μg/ml, respectively). All chicks were given unlimited source of food and water and were raised under identical environmental and management protocols (7, 12). As shown in Table 1, diet was prepared in the form of corn–soya for all groups. The entire starter, growing, and finisher period diets were balanced according to a previous report (13). All chickens were immunized under routine immunization programs for infectious bronchitis and Newcastle and Gumboro diseases. The body weight (BW), weight gain (WG), feed intake (FI), and feed conversion ratio (FCR) were estimated and then compared among groups on days 21 and 42 (7, 12). Moreover, cecal contents from each bird were collected in sterile tubes individually for analysis (14). All methodologies of animal testing were carried out in line with the animal care and use committee of the Veterinary Medicine Faculty, Mansoura University, Mansoura, Egypt. Protocol dated 20032020 gave its approval to the Animal Experimental Guidelines.

**Table 1:** Chemical composition of the diet.

**Ingredients**	**Ration**		
	**Starter**	**Grower**	**Finisher**
Crude protein (%)	23	21	19
Crude fat (%)	5.92	6.62	6.86
Metabolizable energy (kcal/kg)	3,020	3,100	3,200
Crude fiber (%)	3.76	3.46	3.20

### Preparation of IgY

Three hundred fertile eggs from hens, purchased from Alarabia Lell-Alaaf, Quesna, Elmenofia governorate, Egypt, were used for isolation and collection of egg yolk IgY. The water dilution (WD) method was used to purify the IgY from egg yolk. It is one of the best precipitation methods. It is a quick and cost-efficient method for isolating IgY from the entire egg yolk (15). The WD method produces the greatest yield (96%) using a salt precipitation cryo-ethanol treatment, followed by the release of the IgY powder by heating, thereby stabilizing the antibody molecules (16). Hen egg yolk regularly has 8–20 mg/ml of IgY (11). Absorbance at 280 nm wavelength using NanoDrop One apparatus and Pierce^TM^ Chicken IgY Purification Kit (Thermo Fisher Scientific^TM^, New York, NY, USA) were used to determine the IgY concentration. The amount of IgY powder was given in a mixture (0.5 g/kg ration) to the chickens starting from 7 days until the slaughtering time based on our previous reports (7, 10). We thought to include IgY in multiple concentrations (1,500, 3,000, and 4,000 μg/ml) in the ration on day 8 because newly hatched chicks would be fed egg yolk IgY on the first days of hatching until the end of the first week of their lives. Consequently, the amount of IgY introduced to birds would be increased daily based on the diet eaten per kilogram

### Behavioral and Welfare Assessments

All chicks were carefully managed and reared during optimal weather conditions in addition to ensuring a low-stress environment to maximize welfare. The behavior of the birds in each treatment group was recorded using the scan sampling method for 4 days a week between weeks 2 and 6 after hatching. The behavioral assays were performed four times a day, once in the morning (0700 hours), mid-day (1,200 h), afternoon (1,500 h), and evening (2,000 h) for 1-h sessions each (17). Behavior was evaluated by recording the number of chicks spotted performing different behaviors at a 15-s sample interval (instantaneous sampling) using a computerized camera recording system. The cameras (Hikvision, Binjiang District, Hangzhou, China) were fixed, directly overhead, and recorded in real time. Data were stored on Hikvision digital video recorders for the behavioral analysis. A well-defined ethogram was developed to define the behaviors we were studying. For instance, ingestion includes eating (the bird's head is extended toward the feeder, pecking the available food resources) and drinking (the bird's beak is in contact with water in or above the drinker, and the bird appears to be drinking) behavior. Preening (where the bird's beak contacts the bird's plumage), dust bathing (when the bird uses its wings, head, neck, and legs to bathe in the dust), and wing/leg stretching are all examples of body maintenance (the bird extends wing or leg out from the body), as well as head scratching (the bird scratch his head with toes).

### Internal Organ Weight

After slaughtering, the crop, proventriculus, gizzard, and intestine were weighed and calculated as a percentage of dressed carcass weight.

### Blood Analysis

All chickens were slaughtered at 42 days of age by decapitation. The values of packed cell volume (PCV, in percent) were then calculated (18). The hemoglobin (Hb, in grams per deciliter) concentration was estimated using a commercial colorimetric kit from Vitro Scientific Company, Egypt (19). In addition, differential leukocytic counts (DLC, in cells per microliter) were estimated based on the morphology and the ratio of each cell. Lastly, we analyzed the heterophil/lymphocyte (H/L, in percent) ratio as an indicator of stress (20).

### Antimicrobial Bioactivity Assay

The antimicrobial bioactivity assay was achieved as previously described (16) using the liquid broth method. *E. coli* were obtained from the Bacteriology Unit, Egyptian Animal Health Research Institute, El-Dokki, Giza. The mid-logarithmic phase of *E. coli* was collected and resuspended in trypticase soy broth 1% to provide 10^6^-10^7^ colony forming units (CFU)/ml. An equal amount of bacterial suspension and the tested sample were mixed together and incubated in the absence of protein (21–23). The killing power of the IgY concentrations to *E. coli* was calculated using the equation described previously (24). Briefly, ΔLog killing = log_10_ nc – Log_10_ np, where nc and np are CFU per milliliter of mock and treated cells, respectively.

### Nutritive Value of Meat

On day 42, moisture was analyzed by oven drying (25), the ash was analyzed by Muffle Furance (26), the protein was analyzed by the Kjeldahl method (25), the fat was analyzed by Soxhlet extractor (27), and thiobarbituric acid (TBA) was analyzed using the Vyncke method (28).

### Enumeration of *E. Coli* in Breast Meat and Cecal Contents

The *E. coli* counts in breast and thigh meat samples and the cecal contents were evaluated using eosin methylene blue, as previously described (4, 5, 29).

### Statistical Analysis

One-way ANOVA using SPSS software, version 16 (30), was used for the analysis of *E. coli* count in cecal contents and in meat samples. WG, FI, FCR, and the counts of *E. coli* were examined as the quantitative parameters. Relationships between variables were analyzed using the general linear model to reduce the possibility of random factors introducing variability into the data and also to allow for the intervention approach. We performed the mean differences in groups by way of the Turkey test. A comparison of variables was done using non-parametric tests. We used ANOVA testing because, in general, our results were just normally distributed: fixed (treatments) and random (repetitions) effect factors. The mean effects showed significance when the *p*-value was <0.05. Moreover, the chi-square test was used to compare the *E. coli* counts. The *F* distribution had two parameters, the between-groups degree of freedom, *k*, and the residual degree of freedom, *N* – *k*, represented as the following ANOVA formula: (*df*_1_ = *k* – 1, *df*_2_ = *N* – *k*), where *df* is the degree of freedom, *k* is the number of groups, and *N* is the number of observations.

## Results

### Behavioral Activities

There were significant differences in behavioral activity between the groups (Table 2). Supplementation of the poultry diet with IgY powder in concentrations of 3,000 and 4,000 μg/ml had a significant effect on the percentages of scan feeding, drinking, and dust bathing. The addition of IgY powder of 3,000 μg/ml improved the feeding (*F*_(3, 176)_ = 1.256, *p* = 0.0004), drinking (*F*_(3, 176)_ = 1.697, *p* = 0.02), and dust bathing (*F*_(3, 176)_ = 1.392, *p* = 0.002) in comparison with the control group. On the other hand, adding IgY powder at 4,000 μg/ml significantly enhanced the feeding, drinking, and dust bathing (*F*_(3, 176)_ = 1.123, *p* = 0.008; *F*_(3, 176)_ = 1.417, *p* = 0.02; and *F*_(3, 176)_ = 1.257, *p* = 0.002, respectively). Moreover, there was no significant difference in dust bathing among chickens fed 3,000 and/or 4,000 μg/ml IgY, but there was an abundant increase.

**Table 2:** Behavioral observations (mean ± SEM) recorded in various experimental groups.

	**Control**	**1,500 μg/ml IgY**	**3,000 μg/ml IgY**	**4,000 μg/ml IgY**
Feeding	5.24 ± 0.4	8.56 ± 0.3	14.90 ± 0.2**	15.10 ± 0.9**
Drinking	3.32 ± 0.3	2.56 ± 0.2	7.31 ± 0.6*	9.49 ± 0.2*
Preening	3.52 ± 0.9	3.41 ± 0.3	2.39 ± 0.5**	2.11 ± 0.4**
Dust bathing	0.25 ± 0.08	0.62 ± 0.03	0.81 ± 0.12	0.75 ± 0.05
Wing or leg stretching	0.44 ± 0.02	0.25 ± 0.02	0.55 ± 0.03	0.61 ± 0.01
Head scratching	0.11 ± 0.01	0.15 ± 0.02	0.13 ± 0.05	0.12 ± 0.02

*Data were presented as the mean ± SEM. Statistics was done using one-way ANOVA*.

**p <0.01, *p <0.05 (significance level compared to the control group).

### Weight of Internal Organs

There was a considerable influence of IgY on the relative organ weight, as shown in Table 3, with the relative weights of the crop, proventriculus, gizzard, and intestine being significantly greater in birds administered IgY at a concentration of 4,000 μg/ml when dressed carcass weight was considered (*F*_(3, 176)_ = 1.361, *p* = 0.01; *F*_(3, 176)_ = 1.123, *p* = 0.05; *F*_(3, 176)_ = 1.067, *p* = 0.05; and *F*_(3, 176)_ = 1.251, *p* = 0.05, respectively). However, the weights in the groups supplemented with 1,500 and 3,000 μg/ml IgY were relatively the same, with no significant differences compared to the control group.

**Table 3:** Weight of internal organs across the experimental groups.

**Internal organs**	**Control**	**1,500 μg/ml IgY**	**3,000 μg/ml IgY**	**4,000 μg/ml IgY**
Crop	0.23 ± 0.001	0.31 ± 0.001	0.22 ± 0.02	0.35 ± 0.05**
Proventriculus	0.495 ± 0.04	0.523 ± 0.02	0.561 ± 0.08	0.712 ± 0.06*
Gizzard	2.30 ± 0.08	2.34 ± 0.06	2.44 ± 0.03	2.66 ± 0.08*
Intestine	4.54 ± 0.50	4.92 ± 0.60	5.25 ± 0.30	6.08 ± 0.90*

*Statistics (mean ± SEM) was done using one-way ANOVA*.

**p <0.01, *p <0.05 (compared to the control).

### Growth, Performance Rate, and Mortalities

The growth and performance rates of chickens showed that the mean of BW was relatively similar on day 1. The WG, FI, and FCR for all groups were also the same, and there was no significant difference among groups observed (Table 4). At the end of week 3, we could not find any significant difference in the WG of the control chickens compared to those that received 1,500 μg/ml IgY. However, chicks supplemented with 4,000 μg/ml IgY had the highest WG (*F*_(3, 176)_ = 1.468, *p* = 0.005) compared with the controls. Moreover, chickens fed 3,000 and 1,500 μg/ml IgY showed no significant differences. The chickens presented no significant difference in growth among treatment groups by the end of the third week when FI was compared. The FCR for chickens at the end of week 3 was lower than that in the controls (*F*_(3, 176)_ = 1.351, *p* = 0.004) at 4,000 μg/ml IgY. When comparing the total FI at week 6 of the growing stage, we found no significant differences. However, the FCR of chickens supplemented with 3,000 and 4,000 μg/ml at the end of week 6 was slightly lower (*F*_(3, 176)_ = 1.423, *p* = 0.046 and *p* = 0.035, respectively) than that of the control chickens and those supplemented with 1,500 μg/ml of IgY powder. Moreover, our result could not record any significant difference in the FCR for chickens that received 3,000 and 4,000 μg/ml IgY; however, the WG at week 6 was improved (*F*_(3, 176)_ = 1.534, *p* = 0.004) in the group that received 3,000 μg/ml IgY compared with the controls. Furthermore, only the control group recorded an 8.8 mortality percentage. As shown in Table 5, chicks supplemented with 4,000 μg/ml IgY had the highest BW (*F*_(3, 176)_ = 1.328, *p* = 0.000) compared to the controls. In addition, the breast and thigh weights of chickens were the highest when supplemented with 4,000 μg/ml IgY (*p* = 0.007 and *p* = 0.033, respectively) compared to the controls. The breast and thigh ratios relative to the BWs were the highest (9.7 and 7.2%, respectively) in chicks supplemented with 4,000 μg/ml IgY compared to the controls.

**Table 4:** Growing rates in the experimental groups fed different IgY concentrations.

**Item**	**Control**	**IgY concentration**		
		**1,500 μg/ml IgY**	**3,000 μg/ml IgY**	**4,000 μg/ml IgY**
Day 1 BW (g)	48.40 ± 2.08	47.60 ± 2.52	48.30 ± 2.43	48.10 ± 2.05
Week 1 WG (g)	106.80 ± 3.33	106.90 ± 5.55	112.20 ± 3.79	111 ± 4.14
Week 1 FI (g)	93.50 ± 6.88	90.70 ± 6.22	88.70 ± 5.11	87 ± 4.44
Week 1 FCR (g)	0.88 ± 0.11	0.85 ± 0.11	0.79 ± 0.07	0.78 ± 0.02
Week 3 WG (g)	708 ± 26.30	731 ± 24.10	735.60 ± 18.40	751 ± 25.60**
Week 3 FI (g)	866 ± 18.30	861 ± 17.30	841 ± 25.20	836 ± 25.80
Week 3 FCR (g)	1.22 ± 0.08	1.18 ± 0.01	1.14 ± 0.04	1.11 ± 0.03*
Week 6 WG (g)	2,125 ± 153	2,161 ± 166	2,329 ± 181.00**	2,277 ± 142
Week 6 FI (g)	3,860 ± 342	3,885 ± 263	3,716 ± 166.00	3,693 ± 154
Week 6 FCR (g)	1.82 ± 0.11	1.80 ± 0.18	1.60 ± 0.22*	1.62 ± 0.13*
Mortalities (%)	8.8	0	0	0

*Data were presented as the mean ± SEM and evaluated using one-way ANOVA*.

*BW, body weight; WG, weight gain; FI, feed intake; FCR, feed conversion rate*.

**p <0.01, *p <0.05 (significance level compared to the control group).

**Table 5:** Weight of the breast and thigh (in grams and percent, relative to bird weight).

**Item**	**Control**	**1,500 μg/ml IgY**	**3,000 μg/ml IgY**	**4,000 μg/ml IgY**
BW (g)	2,099.60 ± 12.30	2,160.21 ± 183.05	2,243.42 ± 145.91	2,475.40 ± 133.20***
Breast *W* (g)	159 ± 5.32	163 ± 5.78	217 ± 9.40	241 ± 10.22**
Breast (% of BW)	7.57	7.55	9.67	9.74
Thigh *W* (g)	142 ± 4.27	151 ± 4.47	162 ± 5.38	173 ± 6.36*
Thigh (% of BW)	6.14	6.99	7.13	7.23

*Data were presented as the mean ± SEM and evaluated using one-way ANOVA*.

*BW, body weight; IgY, immunoglobulin Y; W, weight*.

***p <0.001, **p <0.01, *p <0.05 (compared to the control group).

### Physiological Analyses

Interestingly, as shown in Table 6, the red blood cell (RBC) counts showed the lowest record (*F*_(3, 176)_ = 1.357, *p* = 0.003) in the group fed 4,000 μg/ml of IgY compared to the control group. However, the Hb concentration recorded no significant difference among groups. Also, the PCV ratio of the group fed 4,000 μg/ml of IgY was the lowest rank (*F*_(3, 176)_ = 1.827, *p* = 0.035) compared to the controls. Meanwhile, the H/L ratio revealed a similarity in all groups. However, heterophils were the lowest (*F*_(3, 176)_ = 1.498, *p* = 0.006), whereas eosinophils were not recorded as significant in the group fed 4,000 μg/ml of IgY. Lymphocytes were more elevated in all IgY-treated groups than in the control group. However, no significant differences were recorded for the basophil and monocyte ratios among all the groups.

**Table 6:** Blood parameters of broiler carcasses fed multiple IgY concentrations at day 42.

**Blood analysis**	**Treatments**			
	**Control (no IgY)**	**1,500 μg/ml IgY**	**3,000 μg/ml IgY**	**4,000 μg/ml IgY**
RBCs (cells/μl)	190 ± 4.32	202 ± 9.44	190 ± 6.88	183 ± 1.66**
Hb conc. (g/dl)	15.11 ± 1.27	15.54 ± 0.98	15.32 ± 0.75	16.19 ± 1.38
PCV (%)	31.66 ± 0.65	33.66 ± 1.23	31.66 ± 0.83	30.5 ± 0.19*
H/L ratio	0.51 ± 0.09	0.94 ± 0.98	1.04 ± 1.21	0.69 ± 1.27
Heterophils (cells/μl)	39.50 ± 4.04	43.42 ± 3.66	42.37 ± 3.74	27 ± 7.54**
Lymphocytes (cells/μl)	57.75 ± 3.25	50.42 ± 3.44	47.75 ± 3.87	64.66 ± 6.77
Basophils (cells/μl)	0.25 ± 0.14	0.14 ± 0.12	0.50 ± 0.18	0.0 ± 0.28
Monocytes (cells/μl)	6.50 ± 1.32	5.85 ± 0.99	8.37 ± 0.98	3.33 ± 0.88
Eosinophils (cells/μl)	1.00 ± 0.61	0.14 ± 0.97	1.00 ± 0.38	0.0 ± 0.14

*Data were presented as the mean ± SEM and analyzed using one-way ANOVA*.

*IgY, immunoglobulin Y; RBCs, red blood cells; Hb, hemoglobin; PCV, packed cell volume; H/L, heterophil/lymphocyte ratio*.

**p <0.01, *p <0.05.

### Assessment of the Antimicrobial Activity of Multiple IgY Concentrations on *E. Coli*

**Figure 2:**
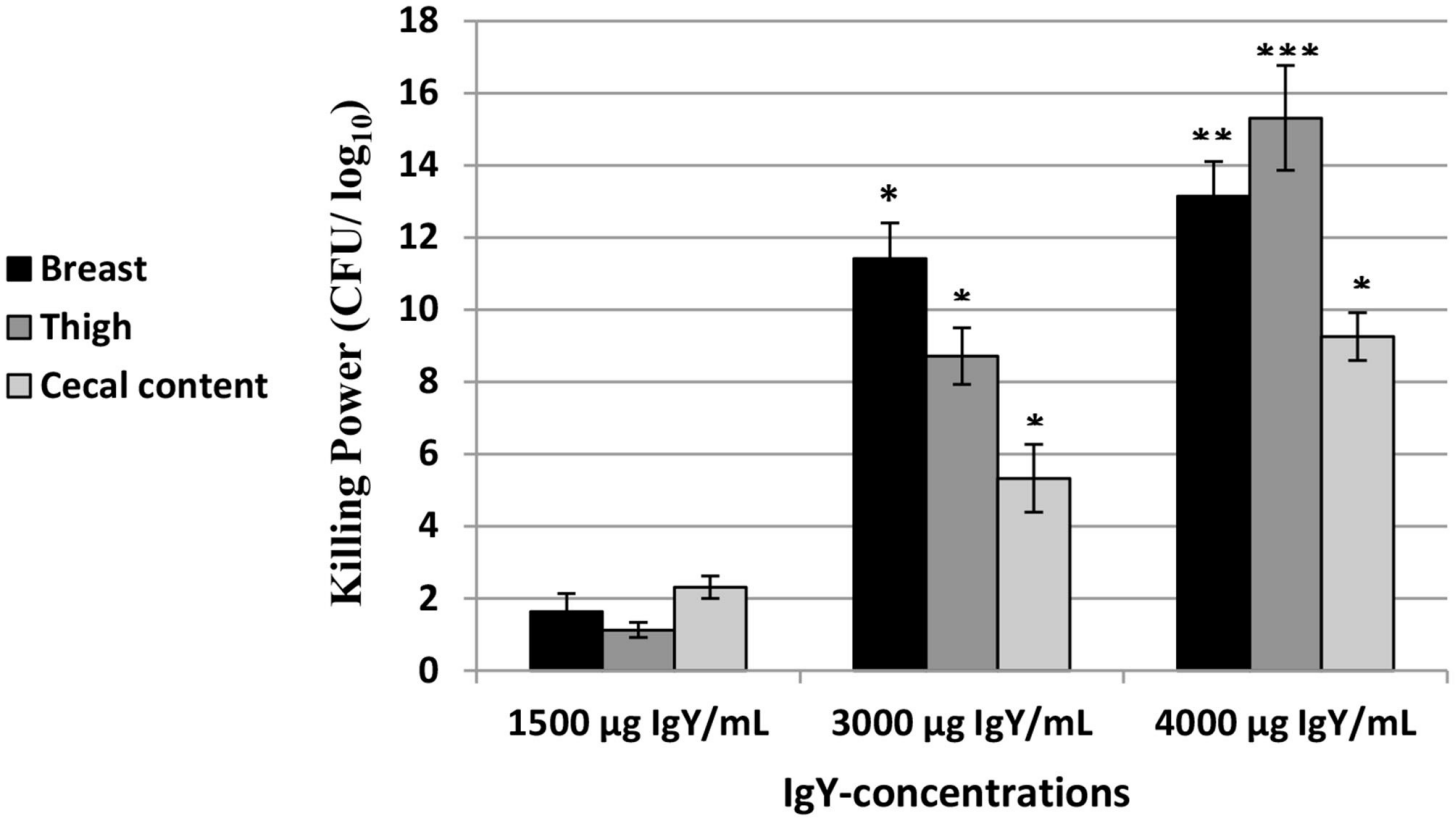
Log_10_ of the antibacterial activity (mean ± SEM) of the immunoglobulin Y (IgY) concentrations (1,500,3,000,and 4,000 μg/ml) in the breast,thigh,and cecal content of chicken against *Escherichia coli*. *p < 0.05,**p < 0.01,***p < 0.001 (compared to 1,500 μg/ml IgY).

As shown in Figure 2, the killing power (Log_10_ CFU/ml) of the concentrations of IgY used (1,500, 3,000, and/or 4,000 μg/ml) was confirmed against *E. coli* in the breast, thigh, and cecal content of chicken. Our results indicated that the highest killing power against *E. coli* is at 4,000 μg/ml of IgY in the breast (*p* = 0.032), thigh (*p* = 0.007), and cecal content (*p* = 0.007) compared to 1,500 μg/ml of IgY.

### Assessment of the Level of Meat Contamination With *E. Coli*

As shown in Table 7, the level of meat contamination with *E. coli* was studied within various groups. Chickens supplemented with 3,000 μg/ml IgY in their rations significantly had reduced levels of *E. coli* contamination in the examined breast and thigh muscles (*F*_(3, 176)_ = 1.527, *p* = 0.046; *F*_(3, 176)_ = 1.328, *p* = 0.039, respectively) in comparison with the control group. Moreover, those fed 4,000 μg/ml IgY had lower levels of *E. coli* contamination in the examined breast and thigh muscles (*F*_(3, 176)_ = 1.527, *p* = 0.031; *F*_(3, 176)_ = 1.328, *p* = 0.025, respectively), comparable to the standard control group.

**Table 7:** *Escherichia coli* counts in meat muscle and cecal contents.

***E. coli* colony count (Log_**10**_ CFU/g)**	**Control**	**1,500 μg/ml IgY**	**3,000 μg/ml IgY**	**4,000 μg/ml IgY**
Breast	7.50 ± 0.20	6.80 ± 0.30	6.20 ± 0.20*	6.10 ± 0.30*
Thigh	6.40 ± 0.30	6.30 ± 0.30	5.80 ± 0.20*	5.70 ± 0.20*
Cecal content	8.31 ± 2.32	7.33 ± 1.82	5.15 ± 1.73*	4.10 ± 1.62*

*Data were presented as the mean ± SEM and evaluated using one-way ANOVA*.

*p <0.05.

### Enumeration of *E. Coli* in the Cecal Content

We found that supplementation of 3,000 and 4,000 μg/ml of IgY in the chicken ration significantly reduced *E. coli* enumeration in the cecum (*F*_(3, 176)_ = 1.679, *p* = 0.028 and *p* = 0.031, respectively). Nevertheless, no significant difference was noticed between bacteria in chicks that received 1,500 μg/ml IgY compared to the controls (Table 7).

### Impact of IgY Concentrations on Meat Quality

As shown in Table 8, the protein and ash percentages were significantly greater (*F*_(3, 176)_ = 1.363, *p* = 0.0001) than those of the control at 4,000 μg/ml IgY. The fat and moisture contents were significantly reduced at 4,000 μg/ml IgY. Interestingly, the TBA value reached the lowest (*F*_(3, 176)_ = 1.529, *p* = 0.028) at 4,000 μg/ml IgY. The quality of broiler meat did not differ significantly between the groups fed 1,500 and 3,000 μg/ml IgY.

**Table 8:** Impact of the different immunoglobulin Y (IgY) concentrations in the broilers' diet on meat quality.

**No**.	**Treatment**	**Nutritive value**				**TBA value (mg/kg malondialdehyde)**
		**Protein (%)**	**Ash (%)**	**Moisture (%)**	**Fat (%)**	
1	Control	18.31 ± 0.12	1.15 ± 0.05	78.10 ± 0.33	2.61 ± 0.01	0.29 ± 0.02
2	1,500 μg/ml IgY	18.90 ± 0.11	1.21 ± 0.04	78.20 ± 0.45	2.32 ± 0.02	0.27 ± 0.02
3	3,000 μg/ml IgY	20.20 ± 0.21	1.57 ± 0.06	75.30 ± 0.51	2.11 ± 0.04	0.25 ± 0.03
4	4,000 μg/ml IgY	21.10 ± 0.27***	1.88 ± 0.05**	73.10 ± 0.34*	2.03 ± 0.05*	0.23 ± 0.01*

*Data were presented as the mean ± SEM and analyzed using one-way ANOVA*.

*IgY, immunoglobulin Y; TBA, thiobarbituric acid; (TBA)*.

***p <0.001, **p <0.01, *p <0.05 (compared to the control group).

## Discussion

The current study determined the optimal concentration of IgY in poultry ration as a potential agent to reduce the contamination level of *E. coli* in meat and intestinal content. The current results indicated that the activity display was increased; these changes in activity could be related to the involvement of IgY in bone health (31). IgY, at special concentrations as a feed additive, can induce many behavioral, physiological, immunological changes and also enhance meat quality. Previously, probiotics used immunomodulation to restore the host's behavior and health (32). However, only a few studies looked at the impact of IgY on chick behavior, performance, and carcass criteria. Our results indicated that IgY powder at 3,000 and 4,000 μg/ml significantly enhanced the percentages of scan feeding, drinking, and dust bathing in comparison to the control group and 1,500 μg/ml IgY. The greater feeding behavior at both 3,000 and 4,000 μg/ml IgY is a result of the positive effect of IgY on the intestinal microflora of birds and its microbiocide effect on pathogenic bacteria. Additionally, this increased the feed use and improved the poultry's health and their meat quality (33). The weights of the viscera and internal organs (heart and liver) were recorded in our previous article (12). Therefore, we confirmed that the IgY-supplemented diet improved the weights of carcasses and internal organs. Herein, we were interested to emphasize the impact of IgY on the digestive system-related organs such as crop, proventriculus, gizzard, and the intestine. Moreover, supplementing the diet with IgY powder at 3,000 and 4,000 μg/ml significantly enhanced the relative proventriculus and intestinal weights compared to those reared under diets of 0 and 1,500 μg/ml IgY. This result indicated that birds fed 3,000 and 4,000 μg/ml IgY had a slight improvement in their body weights.

Furthermore, the current results demonstrated that the drinking activity of chicks was significantly developed in the 3,000- and 4,000-μg/ml IgY groups in comparison to the control and 1,000 μg/ml IgY groups. The great water intake in birds may be attributed to the enhanced feeding activities (34). Also, the current results showed that IgY at different concentrations did not affect chick preening and head shaking activities in comparison to the control. However, broilers fed 3,000 and 4,000 μg/ml IgY had a significant increase in dust bathing and body stretching activities. This improvement in stretching movements may reflect the improvement of the health status of the birds (35).

Various approaches to reduce the microbial contamination of carcasses and meat products can improve the meat quality besides its shelf life (36). The high usage of drugs in the poultry industry leaves accumulating residual effects, and the genetic transfer associated with antibiotic resistance in meat is a serious concern threatening consumers of chicken meat (37). Therefore, natural ingredients in the poultry ration are extremely beneficial to poultry farms for improving public health and biosafety protocols. The expansion of IgY immunization is a strong alternative to antibiotics in poultry feed (38, 39). The antibacterial effect of IgY has been evaluated, according to data provided *in vitro* (40) and in animal health clinical studies (12, 23). The latest studies confirmed the influence of IgY as an active antibacterial agent for Gram-positive bacteria; however, no killing influence had been recorded for Gram-negative bacteria (12). Cytokine activity was highly stimulated by purified IgY compared to other food supplements (41). Moreover, in our study, broiler meat from the group supplemented with IgY showed the most desirable meat quality characteristics, color, shearing force, and water holding capacity (12). Therefore, feeding birds IgY during the production cycle can improve the conversion of their feed (7, 10, 25). We investigated the antimicrobial activity of multiple concentrations (1,500, 3,000, and 4,000 μg/ml) of IgY *in vitro* and discovered that 4,000 μg/ml IgY had bacteriocidal effect against *E. coli*. This finding supported the results of our previous report (12) indicating the therapeutic advantages of IgY against Gram-positive bacteria.

Our findings demonstrated that the mixing of IgY to the diet had little effect on FI until day 42, which agreed with previous reports (7, 12). However, the doses of 3,000 and 4,000 μg/ml IgY significantly improved the WG and FCR on days 21 and 42 compared to the control group. Concerning the impact of IgY on *E. coli* contamination, in the groups fed 3,000 and 4,000 μg/ml of IgY, the levels of *E. coli* in meat and cecal contents were significantly more reduced than in chickens that received the control diet. In addition, the performance and meat quality were improved at all three doses of IgY (1,500, 3,000, and 4,000 μg/ml) on days 21 and 42. In addition, the changes in the gastrointestinal tract microflora seemed to play a big role in lowering meat contamination (42). Hence, IgY, as the primary compound extracted from egg yolks, has proteolytic stability in the stomach and intestines if administered orally (43). Moreover, the stability of IgY in the alimentary tract is dependent on a number of factors involving the pH state and enzymatic activity (44). Consequently, it depends on the health conditions, feeding programs, and the age of chickens. Maternal antibodies are used as food additives for animals since they can proceed with the pelleting processes of the ration throughout the high temperature (45). The activity of an antibody may be improved when prepared at 95°C if particular carbohydrates are present in IgY preparations and/or steaming during the pellet processing to kill bacterial cells (26). While the heat stability of IgY is appropriate for mixing in the ration, we found that its binding activity dropped at extremely high temperatures (46).

In our study, 3,000 and 4,000 μg/ml of IgY led to a great WG and improvement of the FCR in chicken rations without any effects on the FI. Our results further demonstrated that adding 4,000 μg/ml IgY to the diet did influence the FI. The active ingredients in IgY play an essential role in the production and secretion of digestive enzymes (43). However, it requires further investigation whether the improved efficacy of the gastrointestinal tract resulting from IgY intake was linked to a lower intestinal bacterial load. Our results evidenced that birds exposed to IgY at a high concentration (4,000 μg/ml) had an improvement in the final BW and FCR due to high immunization. Such improvements in WG and immunity could be related to the capability of IgY to inhibit the proliferation of pathogenic bacteria and, therefore, improve feed utilization. In our work, the feed efficiency of broilers was not altered by adding 1,500 μg/ml IgY to the ration. The PCV values might change due to the immune response of broilers (25). It was reported that the PCV changed in the case of immune suppression (7, 47).

Herein, the PCV values, RBC counts, and Hb concentrations recorded non-significant differences, which confirmed that different concentrations of IgY supplementation showed no physiological changes. Moreover, the H/L ratio in the group fed 4,000 μg/ml IgY was relatively similar to the control. It revealed that the different concentrations of IgY supplementation failed to change the H/L ratio, which was supported by our previous results (7). However, in the group given 4,000 μg/ml IgY, the numbers of heterophils and eosinophils were lower. It could be caused by the failure of IgY to bind to Toll-like receptor 4 on immune cells, resulting in the cancellation of pro-inflammatory cytokines and the deactivation of heterophils (48). It was noticeable that RBCs, the Hb concentration, the percentage of PCV, and heterophils had the highest values when chickens were fed 1,500 μg/ml IgY but with no significant differences. Then, they returned relatively to normal values of the controls when the birds were fed a double concentration (3,000 μg/ml IgY). It seemed a type of compensatory mechanism that was essential for their static homeostasis. Moreover, we evidenced that the H/L ratio was altered in birds fed variable IgY concentrations due to individual physiological stress. However, there were no significant differences recorded for basophils, monocytes, and eosinophils. Therefore, the stability of the physiological parameters, as referred to above, would help in maintaining the passive immunization of the IgY supplement (49). Interestingly, the circulating IgY of the chicks can be determined by the levels in the plasma of the dam and passed on by the egg yolk (7, 10, 12, 50). The plasma IgY levels were lowered mostly on day 2, as it was expected up to 2,000 μg/ml, as previously indicated (51). Moreover, the IgY transfer from the mother to the chicks occurred on day 4 (1,010 μg/ml), followed by a significant decline on day 7 (830 μg/ml) (10, 28). The occurrence of the IgY half-life was in chick plasma on day 1, whereas it was reduced on days 2 and 3 (52), depending on the stored amount of IgY. Thus, the correlation between circulating IgY from the body of the chick and the level of their activities during their first week of life was strongly positive. Therefore, IgY plays a multidisciplinary role in the activation of early immunity and in chickens' health (53, 54). The disparity most likely occurred due to the wide variety of levels/concentrations of IgY in hens, which was consequently related to the withdrawal amount of IgY during the chick's aging process (51, 52).

The protein content and ash percentage were greater in the group fed 4,000 μg/ml IgY compared to the controls, demonstrating the improved broiler's meat quality in this target group. The higher stability of the fat was recorded in the group fed 4,000 μg/ml IgY due to the minimal TBA values, demonstrating that IgY plays an important role in enhancing the shelf life of broiler meat. Our results agreed with previous reports that discussed the role of the addition of probiotics to improve the TBA values during storage of broiler meat compared to the control group (55, 56).

The level of meat contamination with *E. coli* was lower, suggesting the enhanced hygienic quality of broiler carcasses in the group fed 3,000 and/or 4,000 μg/ml IgY. Using 1,500 μg/ml IgY supplementation was beneficial for improving the physiology of birds and their meat quality and had a bacteriocidal effect on Gram-positive, but not on Gram-negative bacteria. However, using higher concentrations such as 3,000 or 4,000 μg/ml IgY will be extremely beneficial for better meat quality and broad bacteriocidal effects on both Gram-positive and Gram-negative bacteria. We strongly recommend using 3,000 or 4,000 μg/ml IgY supplementation in the diet in large-scale production. These IgY concentrations played an integral antibacterial bioactivity role. Therefore, optimizing the concentration of IgY, a novel future of preventive medicine, reduces *E. coli* contamination and will keep the broilers, as well as consumers, healthy. Taken together, this novel dietary supplement will help achieve the concept of a One Health mission.

## Conclusions

We conclude that supplementation of optimized concentrations of IgY in poultry rations improved the growth rate and the hygienic status of broiler meat. The obtained findings confirmed that the counts of *E. coli* in the meat and intestinal content were lowered by adding optimized concentrations of IgY (3,000 and/or 4,000 μg/ml) in poultry rations. Consequently, it reduced carcass contamination and improved the WG and FCR. Nevertheless, there was no significant impact on *E. coli* reduction after the addition of low concentrations of IgY to the broiler diet. Moreover, 1,500 μg/ml IgY improved the behavioral and physiological conditions of broilers, in addition to the enhanced nutritional benefits to consumers. We strongly recommend using IgY in large-scale poultry production due to its multidisciplinary advantages. Our work, therefore, has great potential in meeting the standard of the One Health assessment, especially regarding animal and human health.

## Data Availability Statement

The original contributions presented in the study are included in the article/supplementary material, further inquiries can be directed to the corresponding authors.

## Ethics Statement

The animal study was reviewed and approved by all methodologies of animal testing were carried out in line with the Animal Care and Use Committee of Veterinary Medicine Faculty, Mansoura University, Mansoura, Egypt, Protocol Dated 20032020 gave its approval to the Animal Experimental Guidelines.

## Author Contributions

IR and AA collaboratively shared for material preparations and the methodologies of this research. For the preparation of scientific paper, AR, MH, MM, and NE developed the hypothesis and concept of the study. GB, AE, MM, WE-G, SA-H, GE-N, BB, MA, MG, AH, and HG participated in the experimental methodologies and analysis, cooperated in the data analysis, writing, and revision of the manuscript. All authors read and approved the final manuscript.

## Conflict of Interest

The authors declare that the research was conducted in the absence of any commercial or financial relationships that could be construed as a potential conflict of interest.

## Publisher's Note

All claims expressed in this article are solely those of the authors and do not necessarily represent those of their affiliated organizations, or those of the publisher, the editors and the reviewers. Any product that may be evaluated in this article, or claim that may be made by its manufacturer, is not guaranteed or endorsed by the publisher.
